# Left Atrial Drainage of the Right Superior Vena Cava: A Case Report

**Published:** 2018-04

**Authors:** Maryam Moradian, Hojjat Mortezaeian, Ramin Baghaei, Behshid Ghadrdoost

**Affiliations:** 1 *Rajaie Cardiovascular, Medical, and Research Center, Iran University of Medical Sciences, Tehran, Iran.*; 2 *Modarres Hospital, Shahid Beheshti University of Medical Sciences, Tehran, Iran.*

**Keywords:** *Heart atria*, *Drainage*, *Vena cava, superior*

## Abstract

An isolated right superior vena cava (RSVC) draining into the left atrium represents a very rare congenital malformation, especially in the absence of a partial anomalous pulmonary venous return. This condition leads to hypoxemia, cyanosis, and clubbing without any other signs of heart defects. We describe an 8-year-old girl, who was referred to our hospital due to unexplained cyanosis. Segmental approach in transthoracic echocardiography showed left atrial drainage of the RSVC, which was subsequently confirmed by contrast echocardiography and angiography. Surgical repair via trans-section and anastomosis of the superior vena cava to the right atrium was performed to prevent the complications of right-to-left shunting and cyanosis. During a 4-year follow-up, the patient remained in very good clinical status and her serial echocardiography was normal except for very mild left atrial and left ventricular enlargement.

## Introduction

The anomalies of the systemic venous return are very rare. Among these, the most frequent is the persistent left superior vena cava (SVC) draining into the coronary sinus. Isolated abnormal drainage of right superior vena cava (RSVC) into the left atrium is very rare, with approximately 20 cases having been reported thus far.^1^^-^^3^ Unexplained cyanosis with different degrees is the most common and sometimes the only clinical manifestation of this extremely rare congenital heart disease, termed “anomalous drainage of the RSVC into the left atrium”. In this anomaly of the systemic veins, the venous blood of the head, neck, and upper extremities returns to the left atrium instead of the right atrium. This anomaly may be diagnosed during the first years of life, but sometimes it is discovered later. This anomaly is likely to beget serious complications like brain abscesses if left untreated.^4^^-^^6^

## Case Report

**Figure 1:**
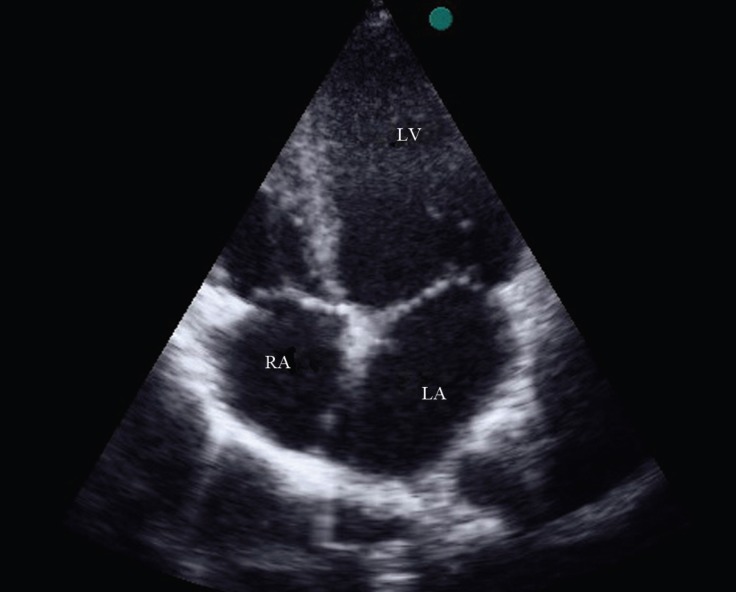
Transthoracic echocardiography in the apical 4-chamber view, showing dilated LV and LA.

LA, Left atrium; LV, Left ventricle; RA, Right atrium

**Figure 2:**
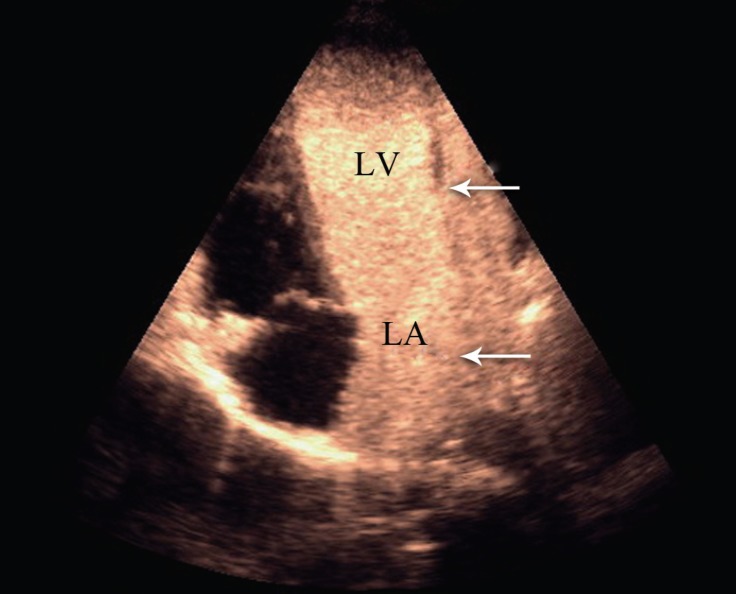
Transthoracic echocardiography in the apical 4-chamber view with contrast study, showing opacification of the left cardiac chambers (arrow heads) after agitated saline injection via the left brachial vein.

LA, Left atrium; LV, Left ventricle

**Figure 3:**
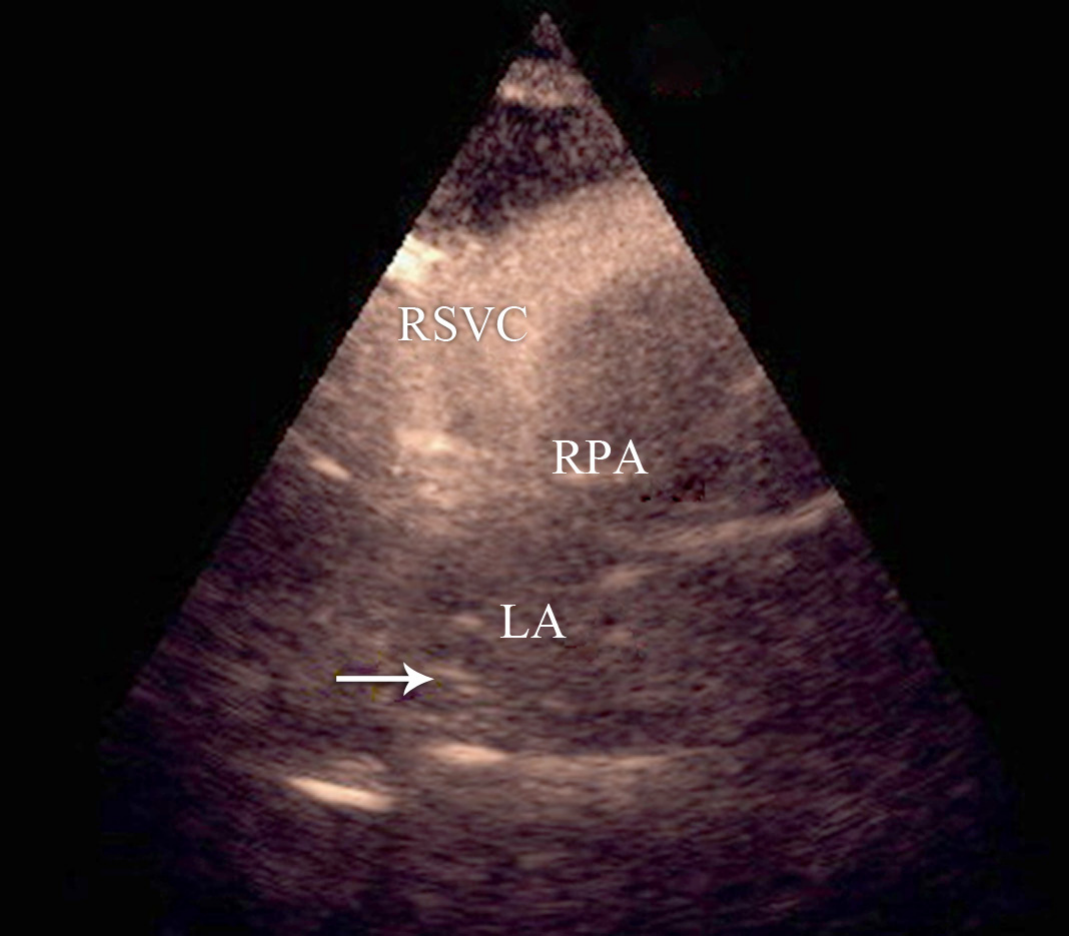
Transthoracic echocardiography in the suprasternal notch view, showing contrast within the dilated RSVC and the entrance of bubbles into the LA (arrow), ruling out an unroofed coronary sinus and a persistent left SVC.

LA, Left atrium; RPA, Right pulmonary artery; RSVC, Right superior vena cava; SVC, Superior vena cava

**Figure 4:**
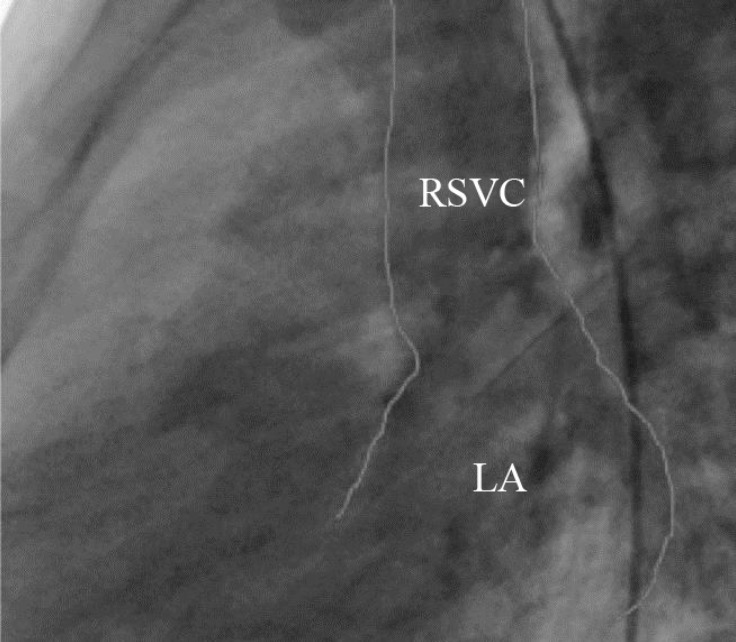
Lateral angiographic projection, illustrating the entrance of the SVC to the LA (posterior ventricle). The outlines of the dilated SVC are shown in white.

LA, Left atrium; RSVC, Right superior vena cava; SVC, Superior vena cava

An 8-year-old girl, who suffered from chronic cyanosis, was referred to our hospital for further evaluation. The patient was well developed with normal growth. On physical examination, a 2/6 systolic heart murmur on the middle left sternal border and mild cyanosis (O_2_ saturation=80% by pulse oximetry) were the only findings. Her blood pressure and heart rate were 110/70 mmHg and 100 bpm, respectively. She had no history of stroke, dyspnea, dizziness, and syncope. Electrocardiography was normal, and there was no finding in favor of the enlargement of any chamber. Chest X-ray showed mild cardiomegaly with normal pulmonary vascular markings. Her hemoglobin and hematocrit levels were 14 g/dL and 41%, respectively. Transthoracic 2D echocardiography, via the segmental approach, demonstrated the normal continuation of the inferior vena cava to the right atrium. However, the orifice of the RSVC to the right atrium could not be visualized. Instead, it seemed that the RSVC was draining into the left atrium. The left atrium and the left ventricle were mildly enlarged (Figure 1). The function of both ventricles was normal. Other echocardiographic findings were mild mitral regurgitation and trivial tricuspid regurgitation. The peak pressure gradient of the tricuspid regurgitation was 20 mm Hg, and there was mild pulmonary valve regurgitation. In addition, there were no intracardiac defects or shunts. The pulmonary veins were connected to the left atrium normally. The diagnosis was confirmed through contrast study with agitated normal saline. Injection via the left brachial vein showed the drainage of the SVC into the left atrium, no atrial septal defect, and no left SVC (Figure 2 & Figure 3). Cardiac catheterization and angiography confirmed a systemic oxygen saturation rate of 80% and a normal pulmonary artery pressure (mean pulmonary artery pressure=15 mmHg). Because transthoracic echocardiography and catheterization confirmed the diagnosis (Figure 4), there was no need to utilize any other imaging modalities.

Surgery was performed through median sternotomy. After aorto-bicaval cannulation and the initiation of cardiopulmonary bypass, cardiac arrest was induced by blood cardioplegia. The SVC was cut from the left atrium, translocated, and anastomosed to the right atrium, which was then augmented with an autologous pericardial patch. The patient was successfully weaned from cardiopulmonary bypass and had an uneventful recovery course.

## Discussion

The persistent left SVC is the most common congenital anomaly of the thoracic venous system. Isolated anomalous drainage of the RSVC into the left atrium is an extremely rare congenital systemic vein malformation, which causes the venous blood return from the head, neck, and upper limbs to drain into the left atrium in lieu of the right atrium.^1^^-^^3^ Patients suffering from this condition tend to have normal growth and development and they are generally asymptomatic. Unexplained cyanosis with different degrees and clubbing are the most common symptoms; older patients, however, may suffer from lightheadedness, chronic headaches, dyspnea, precordial pain on exertion, and easy fatigability or may even experience brain abscesses and stroke.^1^^-^^3^^, ^^7^ A variation of this malformation is the biatrial drainage of the RSVC.^4^

 It seems that that the malposition of the right horn of the sinus venosus toward left with a cephalic direction gives rise to this anomaly during fetal life. Another theory postulates that a seal formed by the fusion of the cephalic portion of the right valve of the sinus venosus and part of the atrial septum superior to the coronary sinus inlet prevents the SVC from draining into the right atrium. This condition is similar to a sinus venosus atrial septal defect in association with the atresia of the SVC oriﬁce and in most cases, the right upper pulmonary vein drains into the RSVC.^1^^, ^^4^^-^^6^ In the prenatal period, fetal echocardiography can detect this anomaly. An enlarged SVC connecting abnormally to a mildly enlarged left atrium can be evaluated in the longitudinal bicaval view.^8^ In the postnatal period, the anomalous connection of the RSVC to the left atrium can be diagnosed via echocardiography, cardiac catheterization, and cardiac magnetic resonance. Nonetheless, sometimes it is just a postmortem finding. 

The most accessible method for detecting this anomaly noninvasively is echocardiography via the segmental approach.^9^^-^^11^ Our patient’s transthoracic echocardiography showed situs solitus, levocardia, d-looped ventricles, and normally related great arteries without any intracardiac defects. There was no visible entrance of the SVC to the right atrium and it seemed to be connected to the left atrium. The left cardiac chambers were dilated, and both left and right ventricular functions were normal. Contrast echocardiography with agitated normal saline showed the immediate appearance of bubbles in the left atrium without opacification of the right atrium.

The appearance of contrast bubbles on the suprasternal notch view ruled out the existence of a left SVC and an unroofed coronary sinus. Our case shows that in the differential diagnosis of hypoxemia, a connection between the SVC and the left atrium should be kept in mind as long as other causes of cyanosis such as cyanotic congenital heart diseases with intracardiac shunts, pulmonary arteriovenous fistulae, abnormalities of the inferior vena cava, and methemoglobinemia can be ruled out.

Surgical correction of this anomaly is necessary because the patient, albeit asymptomatic, is at risk of the complications of right-to-left shunting and hypoxemia, including brain abscesses and paradoxical emboli. During the reconnection of the SVC, it is important not only to ascertain that there is no unrecognized abnormal pulmonary vein drainage but also to prevent any stenosis in the SVC–right atrium connection. Our patient had an uneventful postsurgical course, and her follow-up clinical visits during a 4-year follow-up period were normal. Moreover, yearly echocardiography showed no stenosis in the SVC pathway, but there was still very mild left atrial and left ventricular enlargement with the normal function of both ventricles. 

## Conclusion

We believe that in dealing with unexplained cyanosis in a child, echocardiography as a noninvasive and accessible screening test is invaluable, especially when the segmental approach is used. Additionally, using agitated normal saline as contrast material will help confirm the diagnosis.

Surgical correction of this anomaly is indicated to prevent complications of cyanosis and the risk of systemic embolization. Reconnection of the right superior vena cava to the right atrium is associated with low risk and good long-term prognosis.

## Notes:

***This paper should be cited as: ***Moradian M, Mortezaeian H, Baghaei R, Ghadrdoost B. Left Atrial Drainage of the Right Superior Vena Cava: A Case Report. J Teh Univ Heart Ctr 2018;13(2): 84-87.
